# Single left coronary artery with origin of right coronary artery from left circumflex: a case report

**DOI:** 10.1186/1757-1626-1-355

**Published:** 2008-11-27

**Authors:** Mohammad Shojaie, Ahad Eshraghian

**Affiliations:** 1 Internal Medicine Department, Jahrom University of Medical Science, Iran

## Abstract

**Background:**

A 40-years-old female presented with intermittent chest pain and dyspnea on exertion.

**Case Presentation:**

Electrocardiography showed sinus rhythm with ST-depression in inferior and lateral leads. Subsequent exercise treadmill testing revealed significant ST-depression in V4–V5 and V6 leads. Coronary angiography later showed a single left coronary artery with right coronary artery arising from left circumflex artery, a rare anomaly of coronary arteries. No atheromatous lesion was seen during angiography.

**Conclusion:**

The dignosis of this anomaly is importsnt because the symptoms cannot be differentiated from atherosclerotic coronary artery disease.

## Case presentation

A 40-years-old female was admitted to the hospital with intermittent substernal chest pain and dyspnea. She visited our outpatient clinic because of exacerbation of symptoms in spite of medication. She had no history of systemic disease such as diabetes mellitus or hypertension or hyperlipidemia and no history of cigarette smoking or alcohol drinking. In her family, there was a history of myocardial infarction in her father, and her brother at the age of 33 years old. Physical examination revealed a well nourished female with a blood pressure of 110/80 and a pulse rate of 80 beats per minute. Cardiac auscultation was normal and the lungs were clear. Peripheral pulsation of radial, popliteal, posterior tibial and dorsalis pedis arteries were normal. Chest X-ray showed normal findings.

**Figure 1:**
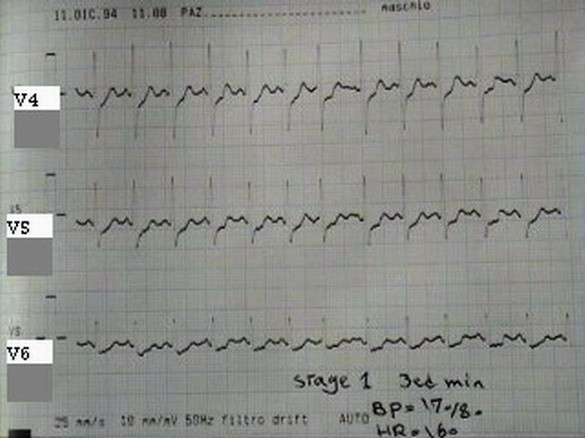
ECG showing 3 mm ST depression in V4–V6.

**Figure 2:**
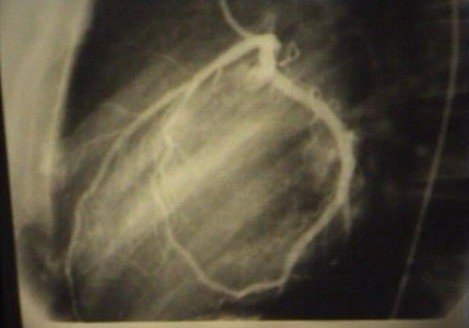
Coronary angiography showing single left coronary artery.

Electrocardiography showed ST-depression in inferior and lateral leads. Echocardiography showed normal left ventricular morphology and no abnormal regional wall motion with ejection fraction of 60%. Exercise treadmill test (Bruce's protocol) was performed that showed significant ST-depression in precordial leads (V4–V6) at the first minute of the test (Fig. 1). At the 4^th ^minute, the test was stopped because of progression of ST-depression to about 3 mm. The patient's laboratory results were normal. Therefore, the patient was referred for coronary angiography. Selective coronary angiography was performed via the right femoral approach (Seldinger technique). Left coronary injection showed normal artery but several attempts for catheterization of right coronary artery was failed. Although aortic root injection was done with suspicion of arterial cut off from the origin, right coronary artery was not appeared. Aortic root angiography showed single left coronary artery and absence of right coronary ostium. Review of left coronary artery revealed anomalous origin of right coronary artery from the left circumflex artery (Fig. 2).

## Discussion

Coronary artery anomaly is a group of diseases with various severities. Yamanaka et al reported coronary artery anomalies in 1.3% of patients in a study of 126595 patients who underwent coronary angiography from 1960 to 1988 at Cleveland clinic [1]. Like our patient most anomalies are discovered incidentally during coronary angiography. Rarely, they produced effort angina, but as you see here effort angina was one of the presenting symptoms in our patient. Coronary anomalies might have clinical consequences such as volume overload, aortic root distortion, bacterial endocarditis and complications during aortic valve surgery.

Anomalous origin of the right coronary artery is a rare congenital anomaly that was 1st described in 1948 by White and Edwards [2]. The prevalence of this anomaly in the white population, as determined from autopsy studies, is 0.026% [3]. The prevalence of this anomaly in other populations, however, is significantly higher. In a study of 13010 patients in Florida, 80(0.61%) patients had coronary anomalies, out of which 50(0.37%) had anomalous origin of right coronary artery with 1 arising from left coronary artery [4]. To our knowledge, there is no Iranian study about the prevalence of right coronary artery anomaly.

Right coronary artery arising from the left circumflex artery in the absence of a normally situated right coronary ostium is considered a variant of single left coronary artery and is a rare phenomenon. Shammas et al reported two cases of a single left coronary artery with continuation of left circumflex artery as the distal right coronary artery without stenotic disease [5]. In a series of 8500 consecutive coronary angiographies, Neuhaus et al reported 3 (0.035%) cases of anatomically single left coronary artery with origin of right coronary artery from the AV branch of dominant circumflex artery in the absence of any coronary artery disease or other cardiovascular abnormalities [6]. Tavernarakis described one case of anomalous origin of right coronary artery from peripheral segment of circumflex artery among 3100 selective angiograms performed in the absence of any clinical abnormality [7]. Ho et al reported a case of anomalous origin of right coronary artery from the left coronary sinus who presented with episodic syncope [8]. Kaul and Javangula described a patient with a single left coronary artery and absent right coronary ostium in whom the right coronary artery has a dual origin. The proximal right coronary artery originated from the left anterior descending and the distal right coronary artery arose as a continuation of left circumflex artery [9].

Our patient was first presented with chest pain and after angiography there was no atherosclerotic lesion found in her coronary arteries. It is well established that an anomalous origin of the right coronary artery can lead to angina pectoris, myocardial infarction, or sudden death, in the absence of atherosclerosis [10].

## Conclusion

Although single left circumflex artery is regarded as a benign condition, we emphasize that the recognition and identification of this anomaly are of clinical importance because the symptoms cannot be differentiated from atherosclerotic coronary artery disease. Among low-risk female patients with chest pain and a positive stress test, coronary artery anomaly should be considered and an angiographic study should be performed.

## Consent

Written informed consent was obtained from the patient for publication of this case report and accompanying images. A copy of the written consent is available for review by the Editor-in-Chief of this journal.

## Competing interests

The authors declare that they have no competing interests.

## Authors' contributions

MS managed the patient, analyzed and interpreted the patient data. AE analyzed and interpreted the patient data and was a major contributor in writing the manuscript.
